# Mechanical and electrical properties of MCMB/Chopped carbon fiber composite with different bead size

**DOI:** 10.1038/s41598-019-43480-4

**Published:** 2019-05-08

**Authors:** Ui-Su Im, Jiyoung Kim, Byung-Rok Lee, Dong-Hyun Peck, Doo-Hwan Jung

**Affiliations:** 1 Department of Advanced Energy and Technology, Korea University of Science and Technology, 102 Gajeong-ro, Yuseong-gu, Daejeon, 305350 Republic of Korea; 2 New & Renewable Energy Research Division, Korea Institute of Energy Research, 217 Gajeong-ro, Yuseong-gu, Daejeon, 34129 Republic of Korea; 3 School of Chemical Engineering, Sungkyunkwan University, 2066 Seobu-ro, Jangan-gu, Suwon-si, Gyeonggi-do 16419 Republic of Korea

**Keywords:** Chemical engineering, Composites, Mechanical properties

## Abstract

The carbonization and graphitization of carbon/carbon (C/C) composites prepared from mesocarbon microbeads (MCMB) and chopped carbon fiber (CCF) have been studied with a wide range of temperatures, CCF contents and MCMB sizes. Three different sizes of MCMB were prepared with coal tar pitch at three temperatures, 420, 430 and 440 °C, and identified as about 12.8, 16.0 and 20.1 µm, respectively. Each size of MCMB was mixed with CCFs at ratios of 2, 4, 6 and 8 wt. % and formed into block shape. After carbonization at 1200 °C, carbonized C/C blocks (CCBs) were graphitized at 2000, 2400 and 2800 °C. The CCB prepared with CCF content of 2 wt. % and an MCMB size of 16.0 µm exhibited the highest flexural strength of about 151 MPa. The graphitized C/C block (GCB) with CCF content of 2 wt. %, which was graphitized at 2000 °C showed the highest flexural strength of about 159 MPa.

## Introduction

Graphite started to be applied in crucibles and pencils in the 15^th^ century. Since the use of carbon electrodes in an electric arc in the 18^th^ century, the demand for graphite has increased, and the manufacturing technology of artificial graphite has been developed^1–3^. Since the Second World War, the aerospace and defense industry has rapidly developed, and studies on artificial graphite blocks with outstanding mechanical, electrical and thermal properties at high temperatures have been actively conducted^4^. Generally, artificial graphite blocks are produced from cokes, binder pitches and impregnation pitches, and the density is improved by repeating the impregnation and carbonization processes^5,6^. This repeating process has the disadvantage of increasing the manufacturing cost of the artificial graphite blocks^7^. On the other hand, mesocarbon microbeads (MCMB) used as a precursor of artificial graphite blocks have an excellent self-sintering property, and are relatively easy to manufacture as an artificial graphite block^8–12^. Furthermore, this block made of MCMB exhibits high mechanical properties without the impregnation process^10,11,13–15^.

To improve the mechanical properties, and the electrical and thermal conductivity of artificial graphite blocks, carbon composites with additives have been developed recently. The additives of carbon composites, such as Si and Ti, have been reported to improve the electrical and thermal conductivity^16–23^. However, such additives have a fatal disadvantage, which is that the dispersion is difficult due to the difference in the density with carbon^24^. To overcome these drawbacks, the research on developing carbon/carbon (C/C) composites has recently been reported by applying crystalline carbonaceous materials as additives^25^. From these reports, it was confirmed that a 3D-ordered carbon structure was established by intercalating additives with a 2D graphite structure, such as CNT, CNF and graphene. Moreover, the 3D-ordered carbon structure improved the mechanical properties and the electrical and thermal conductivity.

C/C composites have excellent mechanical properties, electrical and thermal conductivity and resistivity to chemical and mechanical ablation at elevated temperatures^26–28^. Due to these outstanding properties, C/C composites have been used as core materials in modern industries, such as arc furnaces, cathodes in aluminum electrolysis cells, nozzles, crucibles, brakes and nuclear reactors. C/C composites are prepared using graphite, pitch-based carbon, cokes, or MCMB as a filler^29–31^. In addition, carbon nanotubes (CNTs), carbon nanofiber (CNFs), graphene and chopped carbon fibers (CCFs) are mainly utilized as additives for C/C composite materials^27,32–35^. In particular, CNTs and CNFs have been actively studied as additives for C/C composites, but there are few attempts to use CCFs^6,36,37^.

In this study, C/C composites designed from MCMB and CCFs were studied from three perspectives. First, the effect of the heat treatment temperature on the MCMB size was confirmed. Second, the effects of MCMB sizes and CCF contents on the carbonized carbon block (CCB) were investigated by the flexural strength, density and SEM images. Lastly, the effects of the graphitization temperature and CCF contents on the graphitized carbon block (GCB) were studied. Thus, the optimal MCMB size and CCF content were determined via the crystallinity, density, surface structure, flexural strength and electrical conductivity of the C/C composites.

## Results

### Effect of the heat treatment temperature on the MCMB size

**Figure 1:**
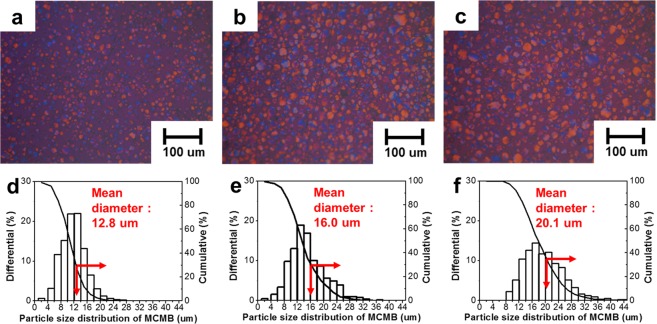
Polarized light micrographs and the MCMB size of the heat-treated coal tar pitch at different temperatures. (**a**,**d**) 420 °C. (**b**,**e**) 430 °C. (**c**,**f**) 440 °C.

**Figure 2:**
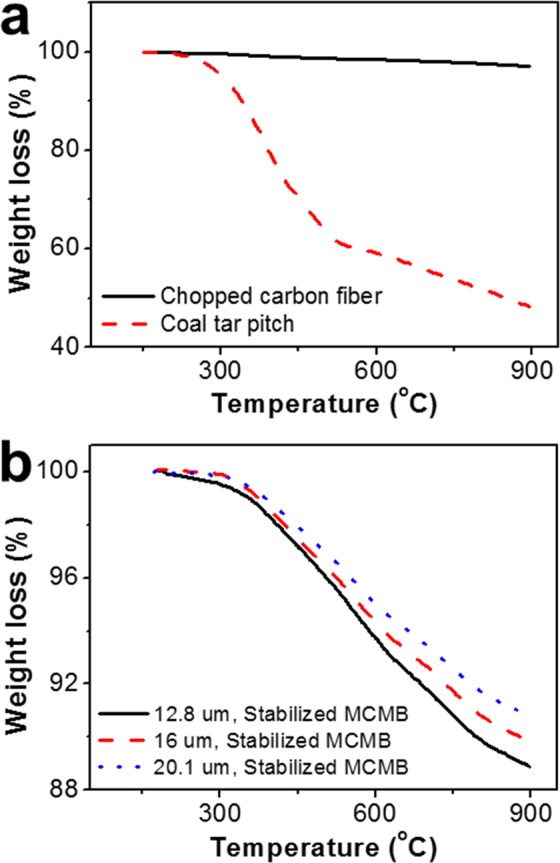
Thermogravimetric analysis. (**a**) the raw materials. (**b**) stabilized MCMBs with different sizes.

The basic properties of the raw materials are listed in Table 1. The length and the diameter of the CCFs were 200 ± 100 µm and 17.6 ± 6 µm. The coal tar pitch contains carbon (92.44%), hydrogen (4.18%), nitrogen (2.88%) and sulfur (0.50%). In Fig. 1a–c, which shows the polarization analysis of the coal tar pitch annealed at 420 °C, 430 °C and 440 °C, it was observed that the size of the mesophase spherules increased as the heat treatment temperature increased. In addition, the yield of the MCMB that were extracted with tetrahydrofuran increased to 53%, 62% and 65%, respectively, as the heat treatment temperature increased. The increase in the yield of the MCMB was thought to result from the growth of the mesophase formation with the rise in the heat treatment temperature^38^. The average size of the MCMB, measured on approximately 500 particles via a microscope, is indicated in Fig. 1d–f. The average sizes of the MCMB prepared at 420 °C, 430 °C and 440 °C were about 12.8 µm, 16.0 µm and 20.1 µm, respectively. The spherical shape of the mesophase spherules was maintained without changes after the extraction and the oxidation stabilization, as displayed in Supplementary Fig. 1^39^. The thermogravimetric analysis of the raw materials is shown in Fig. 2a. Figure 2b shows the volatile content from 100 °C to 900 °C of stabilized MCMB with different sizes. In particular, the fixed carbon of the CCF was about 98 wt. %, and the difference of fixed carbon between CCF and the stabilized MCMB was about 8 wt. %. A volatile matter of MCMB are known as H_2_, CH_4_, CO and CO_2_^40^, and these volatile matter decreased with increasing MCMB size, as shown in Fig. 2b.

**Table 1:** Properties of the raw materials.

Materials	Elemental analysis [wt. %]				Dimension [µm]	
	C	H	N	S	length	diameter
Coal tar pitch	92.44	4.18	2.88	0.50	—	—
Chopped carbon fiber	96.22	0.67	3.09	0.02	200 ± 100	17.6 ± 6

### Effect of MCMB sizes and CCF contents after carbonization

**Figure 3:**
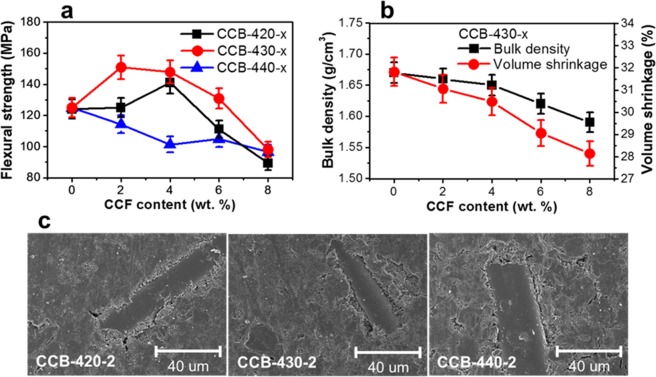
Mechanical and microstructure properties of CCB. Effect of the CCF content after carbonization on (**a**) flexural strength, (**b**) bulk density and volume shrinkage of CCB-430-x. (**c**) SEM photomicrographs of the CCBs prepared with different MCMB sizes.

CCBs were named depending on two factors: the reaction temperature for the mesophase spherules (420, 430 and 440 °C) and the CCF contents (2, 4, 6 and 8 wt. %). For example, CCB-430-2 means that the first heat treatment is at 430 °C and the amount of CCFs is 2 wt. %. The variation of flexural strength in accordance with the CCF content and the size of MCMB is shown in Fig. 3a. The CCB made of a particle size of 16 µm exhibited an excellent flexural strength compared to other MCMB sizes. As shown in Supplementary Table 1, CCB-430-2 and CCB-430-0 were found to have the highest flexural strength of about 151 MPa and the highest density of 1.67 g/cm^3^, respectively. The CCBs prepared from the MCMB of three particle sizes showed the highest bulk density of 1.64 g/cm^3^, 1.67 g/cm^3^ and 1.65 g/cm^3^ in CCB-420-0, CCB-430-0 and CCB-440-0, respectively (Supplementary Table. 1). The volatile contents of 11.1%, 10.2% and 9.2% were presented in the three MCMBs of about 12.8 µm, 16.0 µm and 20.1 µm respectively (Fig. 2b). In addition, the volume shrinkages of CCBs were 31% in CCB-420-0, 31.8% in CCB-430-0, and 30.6% in CCB-440-0 (Supplementary Table. 1). On the other word, the volume shrinkages of CCB was largest in CCB-430-0 even though the volatile contents that can be acted as self-sintering property was highest in CCB-420-0. Thus, due to the relation between the high weight loss and large volume shrinkage, the density of CCBs prepared by MCMB size of 16 µm was higher than the density of CCBs manufactured by other MCMB sizes. Additionally, the optimal CCF content was confirmed to be 2 wt. % in CCB-430-x, and the flexural strength decreased as the CCF content increased. The bulk density and the volume shrinkage decreased with increasing CCF content, as indicated in Fig. 3b. This phenomenon appears to occur because the CCF has a less volatile content than the stabilized MCMB (Fig. 2). From the viewpoint of flexural strength, the flexural strength was remarkably excellent in the CCBs with the CCF contents of 2 and 4 wt. % and decreased as the CCF content exceeded 4 wt. %. These results suggest that the main factor to improve the mechanical properties is the density, and the additional factor is the proper CCF content.

Cracks were produced in CCBs depending on the CCF content, and the surface of CCBs became rough (Supplementary Fig. 2). The CCBs containing CCFs of more than 6 wt. % were found to have an MCMB drop on the boundary surface, which was in contact with the CCFs, and a crack of 50 µm or larger. Figure 3c shows the surface of the CCBs in accordance with the MCMB size. The boundary surface between the CCF and the MCMB was deep and clear in CCB-440-2 fabricated with the MCMB of 20.1 µm, compared to other MCMB sizes, and cracks of 1–10 µm were discovered on the boundary surface between MCMB particles. In addition, as the volume shrinkage decreased, cracks were generated on the surface of the CCBs, and the bulk density and the flexural strength were decreased, as listed in Supplementary Table 1. Thus, the preparation conditions for the CCBs with excellent mechanical properties were demonstrated to be an MCMB size of 16 µm and an CCF content of 2 wt. % via a surface structure.

### Effect of graphitization temperature and CCF contents after graphitization

**Figure 4:**
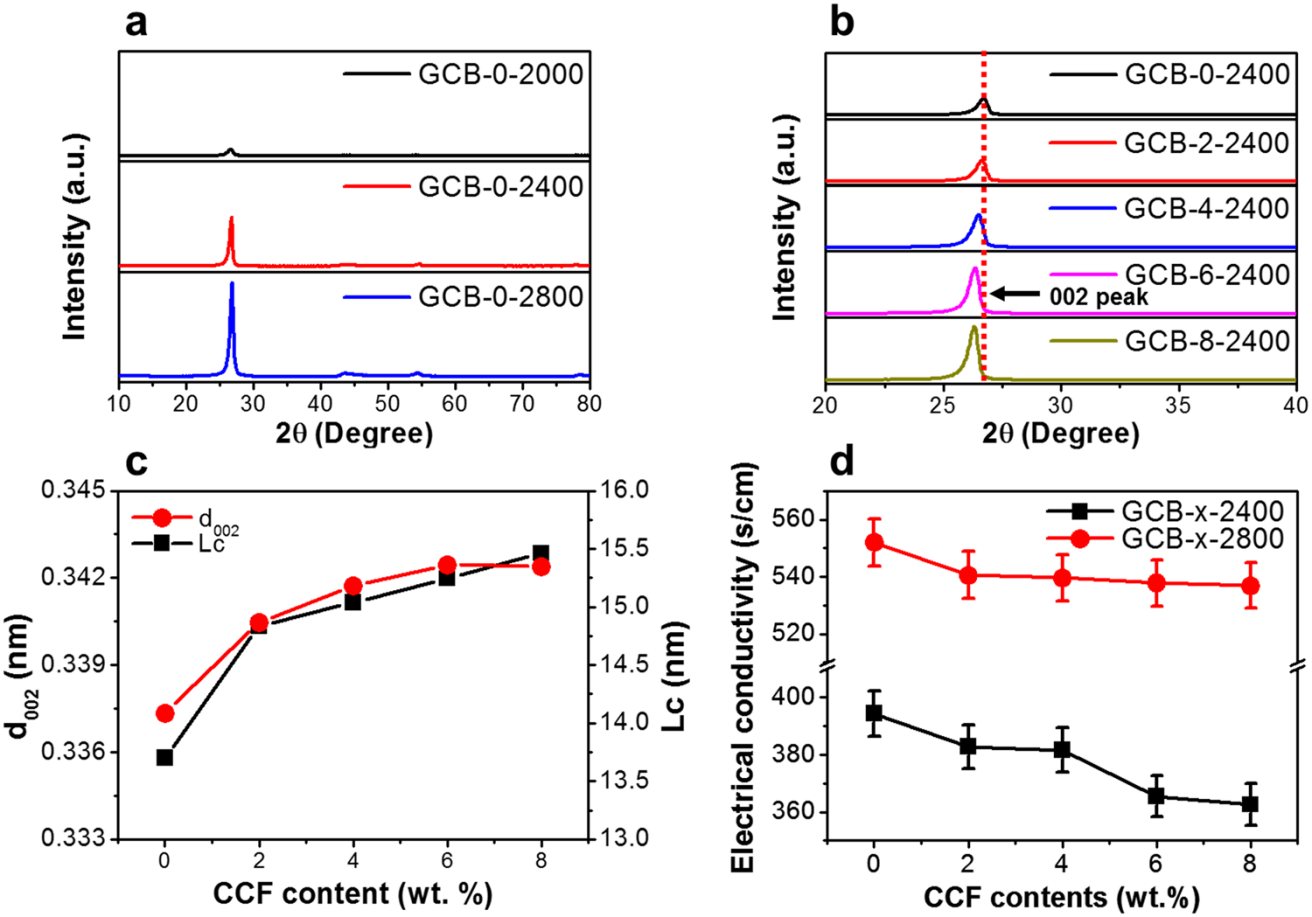
X-ray diffraction patterns and electrical conductivity of the GCB. XRD patterns of GCB (**a**) at different graphitization temperatures and (**b**) different CCF contents. (**c**) Changes in the graphite interlayer spacing d_002_ and the average crystallite thickness Lc as a function of CCF contents after graphitization at 2800 °C. (**d**) Electrical conductivity of the GCB with increasing CCF content.

All the GCBs were prepared from CCB-430-x with the highest mechanical properties. The sample names of GCBs graphitized at different temperatures (2000, 2400, 2800 °C) were classified by adding the graphitization temperature to the sample names. For example, GCB-2-2800 means that CCB-430-2 is graphitized at 2800 °C. As the graphitization temperature increased, the peak of the interlayer spacing (d_002_) shifted to higher angles (Fig. 4a). However, the peak of d_002_ shifted to lower angles with increasing CCF content (Fig. 4b). Figure 4c shows changes in the graphite interlayer spacing d_002_ and the crystallite thickness (Lc) of the GCB at 2800 °C, as a function of the CCF content. d_002_ and Lc increased with the CCF content based on structurally the combination between the fibers and the matrix (Supplementary Fig. 2, Fig. 3c)^6,41^. Thus, the CCF is considered to increase the crystal size easily at the graphitization temperature, and the MCMB tend to have a 2D structure of graphite compared to the CCF^6,41–43^. In Fig. 4d, the electrical conductivity showed a tendency to decrease as the CCF content increased. In addition, as the CCF contents increased, the amount of electrical conductivity reduction was smaller GCB-x-2800 than GCB-x-2400. This is attributed to the increased density and crystallinity by increasing the graphitization temperature^13,24^. The highest electrical conductivity of 552 S/cm was achieved in GCB-0-2800, and all the GCBs graphitized at 2800 °C exhibited an electrical conductivity over 536 S/cm.

**Figure 5:**
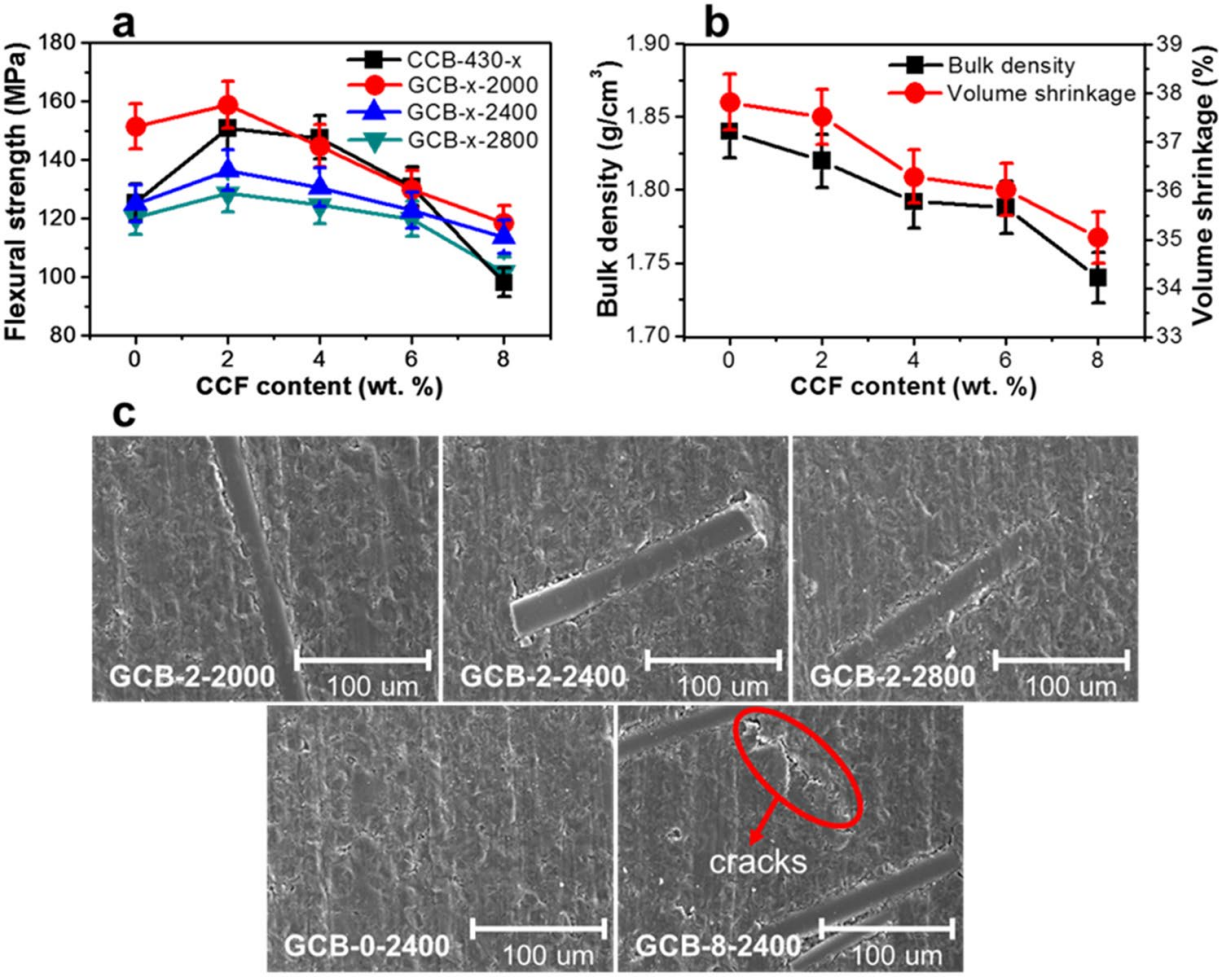
Mechanical and microstructure properties of GCB. Effect of the CCF content on the (**a**) flexural strength, (**b**) bulk density and volume shrinkage after graphitization at 2800 °C. (**c**) Morphologies of the GCBs with different graphitization temperatures and CCF contents.

The flexural strength peaked for the GCB with a CCF content of 2 wt. %. The flexural strength decreased as more than 2 wt. % of CCFs were added (Fig. 5a). The highest flexural strength of about 159 MPa was achieved in the GCB treated at 2000 °C, and the flexural strength decreased with increasing graphitization temperature (Fig. 5a and Supplementary Fig. 3). The bulk density and the volume shrinkage of the GCBs graphitized at 2800 °C decreased with increasing CCF content, as depicted in Fig. 5b. In addition, the bulk density and the volume shrinkage increased with the graphitization temperature (Supplementary Table 2). This is because the graphitization, which changes a turbostratic structure to the 3D-ordered graphite lattice, reduces the interlayer spacing and causes a van der waals force favouring the shear stresses that produce a fracture of GCB^8,44,45^. As described above, the bulk density is an important factor for improving the flexural strength of GCBs, and the addition of 2 wt. % CCFs seems to be another outstanding factor for enhancing the flexural strength.

Cracks were produced on the boundary surface between the CCF and the MCMB by elevating the graphitization temperature over 2000 °C, and the surface of the GCB appeared rough (Fig. 5c)^46–49^. Thus, the reason, why the flexural strength of the GCBs graphitized at 2000 °C is the highest is that the graphitization temperature changes the interfacial structure between the CCF and the MCMB. In Fig. 5c, the GCBs containing 2 and 0 wt. % CCFs exhibited no cracks. Also, the GCBs with 8 wt. % CCF exhibited cracks of approximately 100 µm between the CCFs. It is considered that the cracks generated by adding a lot of CCFs reduced the bulk density and the volume shrinkage^6^.

## Discussion

In summary, C/C composites with excellent mechanical properties were developed from MCMB and CCFs. The dependence of the C/C composite characteristics on the MCMB sizes, CCF contents and graphitization temperature are summarized as follows: (1) As the temperature increased, a mesophase was formed actively and the yield of MCMB increased. (2) The CCBs produced from MCMB with a size of 16 µm exhibited the highest bulk density, and the optimum CCF content for improving the flexural strength is 2 wt. %. (3) With increasing CCF content, the interlayer spacing and the crystal size of the GCBs increase and the electrical conductivity decreases. (4) The increase in the graphitization temperature causes cracks to grow on the boundary surface between the CCF and the MCMB, thereby worsening the mechanical properties. (5) The CCF addition and the bulk density played an important role in manufacturing C/C composites with outstanding flexural strengths.

## Methods

### Preparation of the C/C composites

The coal tar pitch was used to manufacture the MCMB and pitch-based CCF was utilized as additive for C/C composites. The coal tar pitch and the CCFs were obtained from OCI Company Ltd. in Korea and Kureha Chemical Industries in Japan, respectively.

The coal tar pitch was first heated at three temperature points of 420, 430 and 440 °C to fabricate mesophase spherules with different sizes under a nitrogen gas flow, as illustrated in Supplementary Fig. 4. The mesophase spherules existed in the heat-treated coal tar pitch were extracted by tetrahydrofuran at 50 °C for 12 h, and the extracted mesophase spherules are called MCMB. As-prepared MCMB were stabilized at 250 °C for 1 h to maintain the spherical shape, and then CCFs with different contents of 2, 4, 6 and 8 wt. % were mechanically mixed with the stabilized MCMB. To obtain green carbon blocks in two sizes, 15 × 15 × 3 mm and 60 × 10 × 3 mm, the powder mixture was molded under 28 MPa by cold compression, and then the green carbon blocks were carbonized at 1200 °C for 1 h. All of the CCB-430-x samples were heat-treated for graphitization at 2000, 2400 and 2800 °C with a heating rate of 1 °C/min in an argon atmosphere and held for 10 min in order to study the effect of CCF contents at each graphitization temperature.

### Properties and characterizations of the C/C composites

The composition of the CCF and the coal tar pitch was measured using an elemental analysis instrument (EA, TruSpec, LECO Corp., USA). The thermogravimetric analysis (TGA, STA 409 PC, NETZSCH Corp., Germany) was performed in a range of room temperature to 900 °C in a nitrogen flow to examine the thermal behavior of the CCF and the coal tar pitch. The polarization microscopy analysis was conducted by using a polarized light microscopy (PLM, BX51M, Olympus Corp., Japan) to measure the particle diameter of the mesophase spherules. The morphology of the CCB and the GCB was observed by scanning electron microscopy (SEM, JSM-6700F, JEOL Ltd., Japan). The crystallinities of the CCB and the GCB were analyzed using X-ray diffractometry (XRD, RTP 300 RC, Rigaku Corp., Japan). The bulk densities of the CCB and the GCB were calculated based on the measurements of the weight and the dimension. The shore hardness (SH, Type-D, Kobunshi Keiki, Japan) was gauged by the ASTM D 2240 standard. A universal testing machine (UTM, WL2100, WITHLAB Ltd., Korea) was used to measure the flexural strengths after carbonization and graphitization with the following equation^13,50,51^:

1 $${\rm{F}}={\rm{3PL}}/{{\rm{2bh}}}^{{\rm{2}}}$$

where F is the flexural strength, P is the breaking force, L is the span length (30 mm), b is the width (10 mm), and h is the thickness (3 mm).

Electrical conductivity (σ) of the GCBs was determined by a four-point probe tester (DMM7510, Keithley, USA) with a probe spacing of 1 mm at room temperature, from the following equation^52–55^:

2 $${\rm{\sigma }}=1/{\rm{\delta }},{\rm{\delta }}={{\rm{R}}}_{{\rm{S}}}\times {\rm{T}},\,{{\rm{R}}}_{{\rm{S}}}={{\rm{K}}}_{{\rm{a}}}\times {\rm{R}}$$

where δ is the specific resistance, Rs is the sheet resistance, T is the thickness, R is the electrical resistance, and Ka is the correction factor for the size and the thickness of the sample, probe spacing and temperature.

## Supplementary information

Supplementary information
