# Supplementary material for: Mechanical and electrical properties of MCMB/Chopped carbon fiber composite with different bead size
Source: Sci Rep. 2019 May 8;9:7065. doi: 10.1038/s41598-019-43480-4 (PMC6506510; doi:10.1038/s41598-019-43480-4)
Supplement: Supplementary file 1 — Supplementary information [file 41598_2019_43480_MOESM1_ESM.docx]

**Mechanical and electrical properties of MCMB/Chopped carbon fiber composite with different bead size**

Ui-Su Im^1, 2, #^, Jiyoung Kim^3^, Byung-Rok Lee^2^, Dong-Hyun Peck^1, 2^, Doo-Hwan JUNG^1, 2, *^

*^1^ Department of Advanced Energy and Technology, Korea University of Science and Technology, 102 Gajeong-ro, Yuseong-gu, Daejeon 305350, Republic of Korea*

*^2^ New & Renewable Energy Research Division, Korea Institute of Energy Research, 217 Gajeong-ro, Yuseong-gu, Daejeon 34129, Republic of Korea*

*^3^ School of Chemical Engineering, Sungkyunkwan University, 2066 Seobu-ro, Jangan-gu, Suwon-si, Gyeonggi-do 16419, Republic of Korea*

**# First author**

*** Corresponding Author**

Doo-Hwan Jung, PhD

Fuel Cell Research Center

New & Renewable Energy Research Division

Korea Institute of Energy Research (KIER)

Tel 82-42-860-3051

E-mail: doohwan@kier.re.kr

**Keywords**

Mesocarbon microbeads, Chopped carbon fiber, Carbon/Carbon composites, Isotropic graphite, Graphitization

**Supplementary information**

**Figure 1.** SEM images of the stabilized MCMB manufactured at different temperatures: (a) 420 °C, (b) 430 °C and (c) 440 °C.


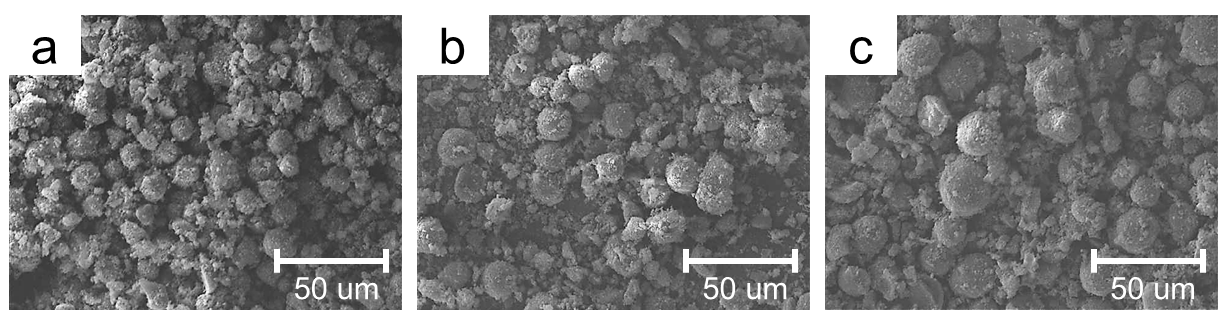


**Figure 2.** SEM images of the CCBs prepared with different CCF contents: (a) CCB-430-0; (b) CCB-430-2; (c) CCB-430-4; (d) CCB-430-6; (e) CCB-430-8.


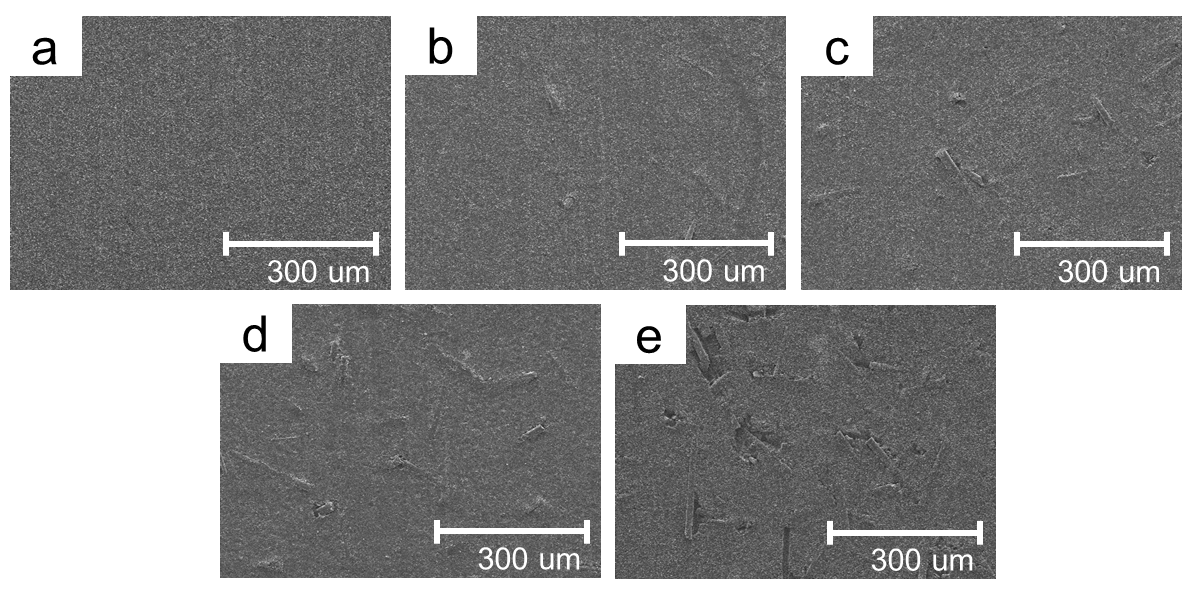


**Figure 3.** Changes in the flexural strength of the C/C composite as a function of the graphitization temperature.


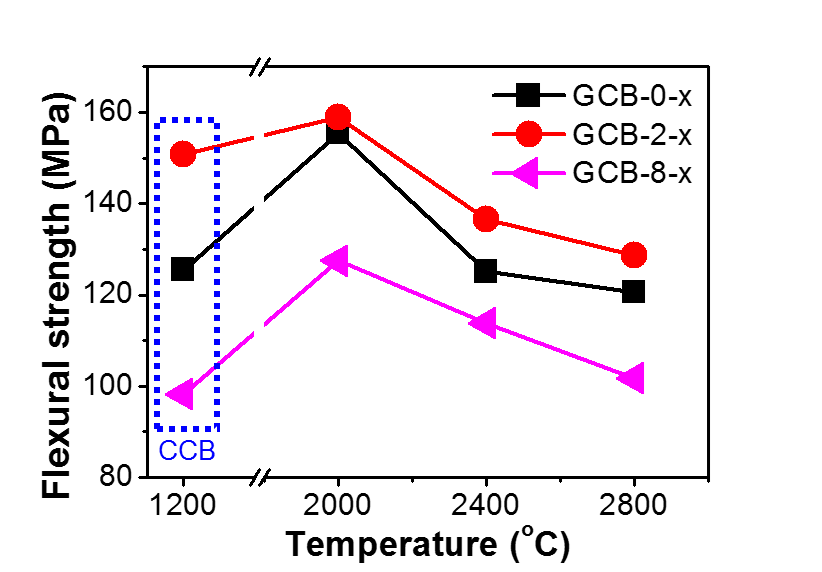


**Figure 4.** Flow diagram for the preparation of C/C composites.


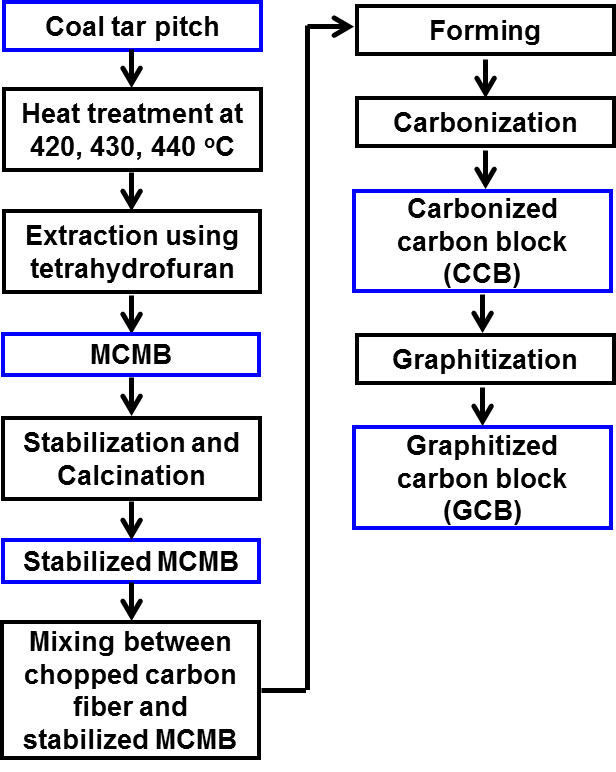


**Table 1.** Physical properties of carbonized carbon blocks at 1200 °C.

| Materials | Shore hardness [HS] | Flexural strength [MPa] | Volume shrinkage [%] | Bulk density [g/cm^3^] |
| --- | --- | --- | --- | --- |
|  |  |  |  |  |
| CCB-420-0 | 94 | 124 | 31.0 | 1.64 |
| CCB-420-2 | 94 | 125 | 30.4 | 1.63 |
| CCB-420-4 | 94 | 141 | 30.1 | 1.62 |
| CCB-420-6 | 93 | 111 | 28.5 | 1.58 |
| CCB-420-8 | 93 | 89 | 26.4 | 1.54 |
| CCB-430-0 | 94 | 125 | 31.8 | 1.67 |
| CCB-430-2 | 94 | 151 | 31.1 | 1.66 |
| CCB-430-4 | 94 | 147 | 30.5 | 1.65 |
| CCB-430-6 | 93 | 131 | 29.1 | 1.62 |
| CCB-430-8 | 93 | 98 | 28.1 | 1.59 |
| CCB-440-0 | 94 | 125 | 30.6 | 1.65 |
| CCB-440-2 | 94 | 114 | 29.9 | 1.63 |
| CCB-440-4 | 94 | 101 | 28.5 | 1.60 |
| CCB-440-6 | 93 | 105 | 28.2 | 1.60 |
| CCB-440-8 | 92 | 96 | 26.5 | 1.57 |

**Table 2.** The volume shrinkage and the bulk density of GCBs.

| Materials | Volume shrinkage [%] | Bulk density [g/cm^3^] |
| --- | --- | --- |
|  |  |  |
| GCB-0-2400 | 37.37 | 1.81 |
| GCB-2-2400 | 36.96 | 1.78 |
| GCB-4-2400 | 36.13 | 1.76 |
| GCB-6-2400 | 35.94 | 1.74 |
| GCB-8-2400 | 34.76 | 1.73 |
| GCB-0-2800 | 37.82 | 1.84 |
| GCB-2-2800 | 37.52 | 1.82 |
| GCB-4-2800 | 36.29 | 1.79 |
| GCB-6-2800 | 36.03 | 1.79 |
| GCB-8-2800 | 35.04 | 1.74 |
